# Lighting up silicon nanoparticles with Mie resonances

**DOI:** 10.1038/s41467-018-05394-z

**Published:** 2018-07-27

**Authors:** Chengyun Zhang, Yi Xu, Jin Liu, Juntao Li, Jin Xiang, Hui Li, Jinxiang Li, Qiaofeng Dai, Sheng Lan, Andrey E. Miroshnichenko

**Affiliations:** 1 Guangdong Provincial Key Laboratory of Nanophotonic Functional Materials and Devices, School of Information and Optoelectronic Science and Engineering, South China Normal University, 510006 Guangzhou, China; 2 School of Physics and Electronic Engineering, Guangzhou University, 510006 Guangzhou, China; 3 Department of Electronic Engineering, College of Information Science and Technology, Jinan University, 510632 Guangzhou, China; 4 State Key Laboratory of Optoelectronic Materials and Technologies, Sun Yat-Sen University, 510275 Guangzhou, China; 5 School of Engineering and Information Technology, University of New South Wales, Canberra, ACT 2600 Australia

## Abstract

As one of the most important semiconductors, silicon has been used to fabricate electronic devices, waveguides, detectors, solar cells, etc. However, the indirect bandgap and low quantum efficiency (10^−7^) hinder the use of silicon for making good emitters. For integrated photonic circuits, silicon-based emitters with sizes in the range of 100−300 nm are highly desirable. Here, we show the use of the electric and magnetic resonances in silicon nanoparticles to enhance the quantum efficiency and demonstrate the white-light emission from silicon nanoparticles with feature sizes of ~200 nm. The magnetic and electric dipole resonances are employed to dramatically increase the relaxation time of hot carriers, while the magnetic and electric quadrupole resonances are utilized to reduce the radiative recombination lifetime of hot carriers. This strategy leads to an enhancement in the quantum efficiency of silicon nanoparticles by nearly five orders of magnitude as compared with bulk silicon, taking the three-photon-induced absorption into account.

## Abstract (web summary)

As an indirect semiconductor, silicon shows notoriously inefficient luminescence. Here, the authors utilize the Mie resonances in silicon nanoparticles to demonstrate visible white-light emission, both from free-standing spheres and particles etched on a silicon-on-insulator substrate.

## Introduction

Being one of the most important semiconductors, silicon has been widely utilized to fabricate electronic devices, waveguides, detectors, solar cells, etc^1–3^. Owing to the existence of electric and magnetic dipole (ED and MD) resonances in the visible to near infrared spectral range, high-index dielectric nanoparticles such as silicon nanospheres (NSs) and nanopillars (NPs) with feature sizes ranging from 100 to 300 nm are considered as promising building blocks for the next-generation metamaterials operating at optical frequencies^3–5^. So far, the linear optical properties of silicon nanoparticles have been extensively studied^2–18^ and much attention has been paid to the nonlinear optical responses of silicon nanoparticles by exploiting the strong field enhancement at the MD resonances^19–26^. Very recently, it was shown that the MD resonances of gallium arsenide NSs can be used to generate efficient hot-electron intraband luminescence^27^, which was previously observed in single plasmonic hot spots^28^.

There is a common belief that silicon is not suitable for making good emitters owing to the low quantum efficiency of bulk silicon (~10^−7^). Although efficient visible light emission from silicon quantum dots with sizes smaller than 10 nm has been demonstrated^29^, they are difficult to be integrated with silicon nitride waveguides. So far, all-Si-based emitters working in the visible light spectrum, which can be integrated to silicon nitride-based circuits^30^, has not been realized. It should be noted that blackbody radiation in the visible to near infrared spectral range, which was observed in silicon nanoparticles of ~100 nm in diameter pumped by high-power laser, is induced by high lattice temperature^31^.

Physically, the quantum efficiency of a material is determined by the ratio of the radiative lifetime (*τ*_r_) to the nanoradiative lifetime (*τ*_nr_), i.e., *η* = 1/(1 + *τ*_r_/*τ*_nr_). In previous studies, much effort has been devoted to the reduction of *τ*_r_ by utilizing the so-called Purcell effect. However, it should be kept in mind that the quality factor of a cavity required to achieve a significant reduction of *τ*_r_ is generally too large to be realized for a subwavelength nanoparticle in the visible light spectrum. Alternatively, it is noticed that the enhancement of *η* can also be achieved by enlarging *τ*_nr_. In silicon quantum dots, it has been shown that the combination of the phonon bottleneck and Auger effect can increase *τ*_nr_ by two orders of magnitude (from 0.1−1.0 ps to 10−100 ps), leading to the observation of the emission induced by phononless interband transition^32,33^. For silicon nanoparticles without obvious phonon bottleneck effect, a dramatic increase of *τ*_nr_ can be realized by exploiting the Auger effect significantly enhanced by the high-density carriers injected through resonantly exciting the MD/ED resonances. In addition, a reduction of *τ*_r_ is expected at the magnetic and electric quadrupole (MQ and EQ) resonances where the electric field is enhanced. Therefore, a significant enhancement of *η* can be achieved by enlarging *τ*_nr_ with the MD and ED resonances and reducing *τ*_r_ with the MQ and EQ resonances, simultaneously.

In this paper, we report the realization of the two-photon-induced and three-photon-induced luminescence generated from silicon NSs and NPs with a feature size of ~200 nm, which act as cavities and antennas simultaneously, by using femtosecond laser pulses with ultralow low energy of ~40 pJ.

## Results

### Field enhancements at resonances and principle

**Fig. 1:**
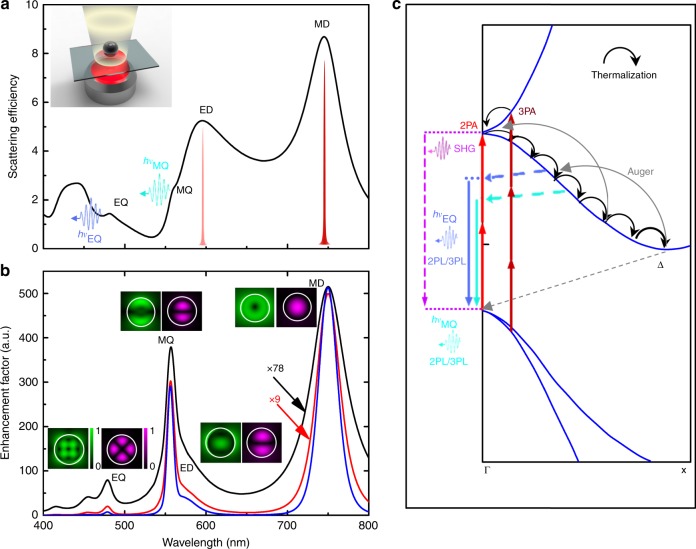
Mechanism for lighting up silicon nanoparticles. **a** Principle of utilizing the magnetic dipole (MD) and electric dipole (ED) resonances of a silicon nanosphere (NS) to realize the excitation enhancement in two-photon-induced absorption (2PA) and three-photon-induced (3PA) and the magnetic quadrupole (MQ) and electric quadrupole (EQ) resonances to realize the emission enhancement in two-photon-induced luminescence (2PL) and three-photon-induced luminescence (3PL). The inset shows schematically the excitation of a silicon NS placed on a quartz substrate by using femtosecond laser light (red color) and the emission of up-converted luminescence (white light). The pulse-like symbols represent the wavelengths of the excitation laser which are resonant with the ED and MD resonances. **b** Spectra of *I*, *I*^2^, and *I*^3^ calculated for a silicon NS with a diameter *d* = 192 nm. The electric (left) and magnetic (right) intensity distributions calculated at the MD, ED, MQ, and EQ resonances are presented as insets. **c** Energy band diagram of silicon in which the carrier excitation process through 2PA/3PA, the Auger effect that continuously lifts carriers from low-energy states to high-energy states, and the photon emission processes through second harmonic generation (SHG) and 2PL/3PL are schematically depicted

We fabricated silicon NSs by using femtosecond laser ablation and regularly arranged silicon NPs by using electron beam lithography and reactive ion etching (see Methods). The crystalline phase of the silicon NSs was characterized by transmission electron microscopy (see Supplementary Note 1 and Supplementary Fig. 1). In Fig. 1a, we present the scattering spectrum of a silicon NS with a diameter (*d*) of 192 nm, which can be decomposed into the contributions of MD, ED, MQ, and EQ resonances (see Supplementary Note 2 and Supplementary Fig. 2), calculated by using Mie theory. Basically, the two-photon-induced luminescence (2PL) intensity of a silicon nanoparticle can be described as follows^34,35^:

1 $$\begin{array}{lcl}I_{2{\mathrm{PL}}} &= \eta (\lambda _{{\mathrm{em}}})\delta (\lambda _{{\mathrm{ex}}})\left| {E_0} \right|^4L^4(\lambda _{{\mathrm{ex}}})L^2(\lambda _{{\mathrm{em}}})\\ &\propto \eta (\lambda _{{\mathrm{em}}})\delta (\lambda _{{\mathrm{ex}}})\left| {E_0} \right|^4\bigg(\frac{1}{V}{\int}_{{\mathrm{Silicon}}} {\left| {E(\lambda _{{\mathrm{ex}}},r)/E_0} \right|^4{\mathrm{d}}V\bigg)}\\ &\quad\times\bigg(\frac{1}{V}{\int}_{{\mathrm{Silicon}}} {\left| {E\left(\lambda _{{\mathrm{em}}},r\right)/E_0} \right|^2{\mathrm{d}}V\bigg)},\end{array}$$

where *η* is the quantum efficiency, *δ* is the cross-section of two-photon-induced absorption (2PA), *L*(*λ*_ex_) = |*E*(*λ*_ex_,*r*)/*E*_0_| and *L*(*λ*_em_) = |*E*(*λ*_em_,*r*)/*E*_0_| are the electric field enhancement factors at the excitation (*λ*_ex_) and emission (*λ*_em_) wavelengths, respectively. By replacing $\frac{1}{V}{\int}_{{\mathrm{Silicon}}} {\left| {E(\lambda _{{\mathrm{ex}}},r)/E_0} \right|^4{\mathrm{d}}V}$ with $\frac{1}{V}{\int}_{{\mathrm{Silicon}}} {\left| {E(\lambda _{{\mathrm{ex}}},r)/E_0} \right|^6{\mathrm{d}}V}$ in Eq. (1), one can obtain the formula describing the three-photon-induced luminescence (3PL) intensity qualitatively. In Fig. 1b, we present the effective spectra of$I^3 = \frac{1}{V}{\int}_{{\mathrm{Silicon}}} {\left| {E(\lambda ,r)/E_0} \right|^6{\mathrm{d}}V}$, $I^2 = \frac{1}{V}{\int}_{{\mathrm{Silicon}}} {\left| {E(\lambda ,r)/E_0} \right|^4{\mathrm{d}}V}$, and $I = \frac{1}{V}{\int}_{{\mathrm{Silicon}}} {\left| {E(\lambda ,r)/E_0} \right|^2{\mathrm{d}}V}$ calculated for the single silicon NS. The electric and magnetic field distributions at the first four resonances are also provided. It can be seen that the enhancement factor for *I*^2^ (or *I*^3^) is more than 50 (or 500) at the MD resonance. In addition, the enhancement factor for *I* is ~5 at the MQ resonance. It implies that an enhancement in 2PL (or 3PL) of more than two (or three) orders of magnitude can be achieved if one excites the silicon NS at the MD resonance and detects the 2PL (or 3PL) at the MQ resonance. Although the energy of one photon at the MD resonance (~758 nm) is not sufficient to induce the direct transition to the conduction band at the Γ point, the large enhancement in |*E*|^4^ (or |*E*|^6^) at the MD resonance would result in a significant enhancement in the 2PA or three-photon-induced absorption (3PA), generating a large carrier density (~10^20^ cm^−3^) in the conduction band (see Supplementary Note 3 and Supplementary Figs. 3−11). In fact, the population of the conduction band through 2PA can be assisted by Rabi oscillation or phonons^36^ and the 3PA process may become dominant at high excitation intensities. As schematically shown in Fig. 1c, the Auger effect, which has been significantly enhanced by the large carrier density, continuously lifts carriers from low-energy states to high-energy states and dramatically enlarges the relaxation time. On the other hand, the interband transitions assisted by phonons preferentially occur at the EQ/MQ resonances when hot carriers relax from the Γ valley to the Δ valley due to the enhanced radiative recombination rates. The combination of these two effects leads to the white-light emission from silicon nanoparticles. Therefore, the physical mechanism for boosting up the photon emission from silicon nanoparticles is substantially different from that reported previously in silicon nanowires utilizing lossy plasmonic effect^36^. In our case, the electric field enhancements at both the excitation and emission wavelengths are mediated by the electric and magnetic resonances in all-silicon nanostructures^3^.

### Nonlinear optical responses of silicon NSs

**Fig. 2:**
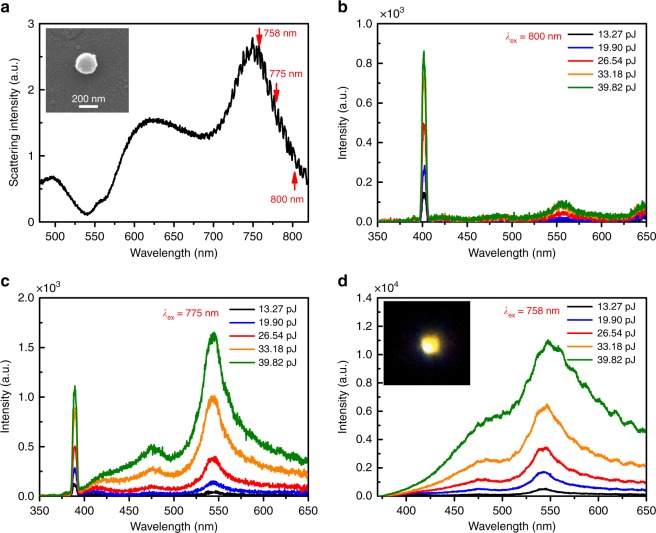
White-light emission from silicon nanospheres. **a** Scattering spectra measured for a silicon nanosphere (NS) with *d* ~192 nm. The inset shows the scanning electron microscope image of the silicon NS. **b**–**d** show the dependence of the nonlinear response spectrum of the silicon NS on the excitation pulse energy measured at 800, 775, and 758 nm, respectively. White-light emission recorded by a charge coupled device is shown in the inset of **d**

In Fig. 2a, we show the measured scattering spectrum for a silicon NS with *d* ~192 nm. We varied the excitation wavelength (*λ*_ex_) and examined the nonlinear optical response. For *λ*_ex_ = 800 nm, we observed strong second harmonic generation at 400 nm and very weak up-converted luminescence peaking at ~556 nm, as shown in Fig. 2b. When the excitation wavelength *λ*_ex_ was tuned to 775 nm, a significant increase of the up-converted luminescence was observed at the MQ and EQ resonances (Fig. 2c). For resonant excitation (*λ*_ex_ = 758 nm), the up-converted luminescence was further enhanced by nearly one order of magnitude and the second harmonic generation was greatly suppressed. In this case, white-light emission could be achieved by using pulse energy as small as ~40 pJ (Fig. 2d) while no signal was detected in the bulk silicon case under the same excitation conditions. The color picture shown in the inset of Fig. 2d and the broad luminescence spectrum demonstrate clearly that the white-light emission originated from the interband transition of hot carriers in a silicon NS can be significantly enhanced by resonantly exciting the MD resonance. This conclusion is validated by other experiments (see Supplementary Note 4 and Supplementary Figs. 12, 13).

### Emission enhancement and luminescence lifetime

**Fig. 3:**
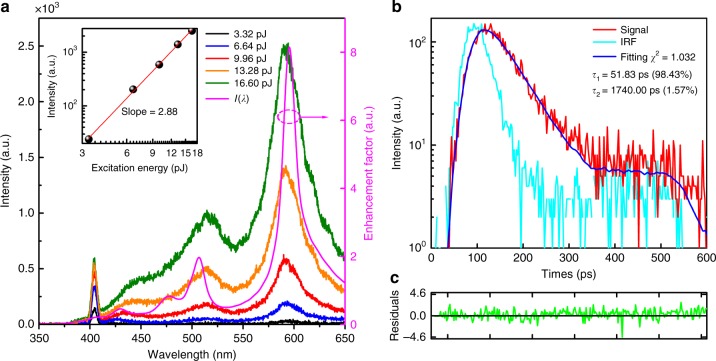
Emission enhancement and luminescence lifetime. **a** Nonlinear response spectra measured at different excitation pulse energies for a silicon nanosphere (NS) with *d* ~210 nm. The spectrum of *I* calculated for the silicon NS is also presented for comparison. The inset shows the dependence of the up-converted luminescence on the excitation pulse energy plotted in a double-logarithmic coordinate. **b** Decay of the up-converted luminescence measured for a silicon NS with *d* ~190 nm after the excitation of the femtosecond laser pulses. The luminescence lifetime is derived to be ~52 ps based on a reconvolution fitting analysis. Here, IRF represents instrument response function and *χ*^2^ is a parameter characterizing the fitting quality. **c** The corresponding residuals for the fitting

In Fig. 3a, we present the nonlinear response spectra of the silicon NS with *d* ~210 nm (see also Supplementary Note 4 and Supplementary Fig. 14). The spectrum of *I* is also provided for comparison. It is found that the peaks in two spectra almost coincide, verifying the enhanced radiative recombination rates achieved at the MQ and EQ resonances. From the dependence of the up-converted luminescence on the excitation pulse energy plotted in the inset, a slope close to 2.9 was extracted, indicating that the up-converted luminescence is dominated by 3PL (see another example in Supplementary Fig. 15). In order to confirm that the hot luminescence from silicon NSs originates from the interband transition of hot carriers, we calculated the dependence of the extracted slope on the wavelength (or energy) of the emitted photon (see Supplementary Note 4 and Supplementary Fig. 16). The extracted slope for excitation wavelengths longer than 730 nm exhibits a constant close to 3.0 over the entire emission spectrum except at the EQ/MQ resonance where a slightly reduced slope is observed. The reduced slopes observed at the EQ/MQ resonances imply enhanced photon conversion efficiencies in the up-converted luminescence at these wavelengths. This behavior is clearly distinct from the hot-electron intraband luminescence observed very recently in single plasmonic hot spots and gallium arsenide NSs where a linear dependence of the extracted slope on the energy of the emitted photons was found^27,28^. The different nature of the band structure between silicon and gallium arsenide is responsible for the distinct hot luminescence observed in silicon and gallium arsenide NSs.

The radiative lifetime for the direct interband transition in bulk silicon (i.e., from the conduction band minimum at the Γ point to the maximum of the valence band) was observed to be 10−100 ns, although very few reports were found for this data^32,37^. This value is almost two orders of magnitude larger than the radiative lifetime for the indirect interband transition in bulk silicon (i.e., from the conduction band minimum at the Δ point to the valence band maximum). From the enhancement factors of electric field estimated for the EQ/MQ resonances (see Fig. 3a), it is expected that *τ*_r_ can only be reduced by about one order of magnitude from 10−100 ns to 1−10 ns. Although it is anticipated that *τ*_nr_ can be significantly increased by Auger effect enhanced by Mie resonances, its value is still much smaller than *τ*_r_. Thus, the decay of the luminescence is dominated by *τ*_nr_ (i.e., *τ*~*τ*_nr_). In Fig. 3b, we show the luminescence decay of a typical silicon NS with *d* ~190 nm measured at ~540 nm where the luminescence is enhanced by the MQ resonance. The luminescence decay is governed by the nanoradiative lifetime (i.e., relaxation time) which was found to be ~52 ps (see another case in Supplementary Fig. 17). It implies that *τ*_nr_ was increased from 0.1−1.0 ps to ~50 ps. Therefore, the quantum efficiency of the silicon NS is expected to be enhanced by four to five orders of magnitude, which is in qualitatively agreement with the value (~1.22%) obtained in measurements of the quantum efficiency after taking the 3PA into account (see Methods and Supplementary Note 3). It should be emphasized that the quantum efficiency measured for a silicon NS depends on the quality of the silicon NS, the excitation wavelength, and the excitation intensity (see Supplementary Figs. 9, 10). In addition, it is noticed from Eq. (1) that the hot luminescence of the silicon NS depends not only on the quantum efficiency but also on the absorption cross-section which can be greatly enhanced by resonantly exciting the MD resonance.

### Nonlinear optical responses of silicon NPs

**Fig. 4:**
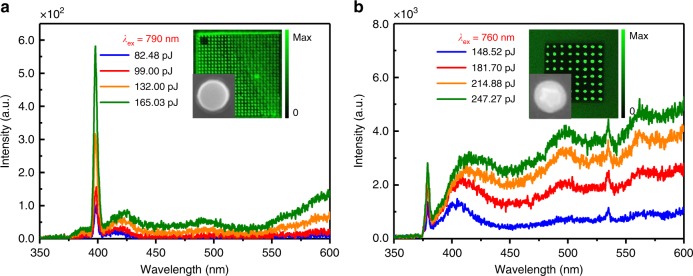
White-light emission from silicon nanopillars. **a** Nonlinear response spectra measured for a silicon nanopillar (NP), which is excited at the magnetic dipole resonance, at different pulse energies. **b** Nonlinear response spectra measured for a single truncated silicon nanocone (NC), which is excited at the electric dipole resonance, at different pulse energies. The insets show the emission patterns of the array of silicon NPs obtained by using a laser scanning confocal microscope. In each case, the scanning electron microscope image for a single silicon NP or NC is also provided as an inset

Although arrays of regularly arranged silicon NSs have been successfully fabricated by using femtosecond laser ablation^9^, it is highly desirable that silicon nanoparticles emitting strong visible light can be achieved by using the state-of-the-art fabrication technologies of semiconductor nanostructures^38^. Therefore, silicon NPs are considered as the most suitable alternative and various functional devices composed of silicon NPs have been experimentally demonstrated^3,6,14–17,19–25^. We also observed up-converted luminescence from silicon NPs fabricated directly on a silicon-on-insulator (SOI) wafer when we excited the silicon NPs at their MD or ED resonances (see Fig. 4, Supplementary Note 5 and Supplementary Figs. 18−20). The confocal microscopic images of the 3PL for the array of silicon NPs shown in the insets of Fig. 4 validate that we did achieve on-chip all-silicon photon source, which is compatible with the current semiconductor fabrication technology. The silicon NPs can also be transferred to a quartz substrate (see Supplementary Note 6 and Supplementary Figs. 21–22). The inefficient white-light emission observed for the silicon NPs on the SOI is mainly caused by the large number of non-radiative recombination centers introduced in the silicon NPs during the fabrication process. Further improvement in hot luminescence can be expected by engineering the electromagnetic modal properties of silicon nanoparticles.

## Discussion

We have proposed and demonstrated a strategy for lighting up silicon nanoparticles by exploiting their Mie resonances. Our integrated silicon nanoemitters are based on all-dielectric materials functioned as cavities and antennas simultaneously and do not employ any lossy plasmonic elements. The white-light emission can be achieved not only in silicon NSs but also in silicon NPs fabricated directly on SOI, which are compatible with the current fabrication technology of Si-based chips. The quantum efficiency is enhanced by nearly five orders of magnitude as compared with bulk silicon, taking the 3PA into account. Our finding might be a solid step toward the achievement of on-chip light sources for silicon nitride-based optical circuits^30^. It might also facilitate other applications in silicon photonics^38^.

## Methods

### Fabrication of silicon nanoparticles

We employed femtosecond laser ablation to fabricate silicon NSs with diameters ranging from 150 to 250 nm. In experiments, 800-nm femtosecond laser light delivered by a femtosecond amplifier (Legend Elite, Coherent) with pulse duration of 90 fs and repetition rate of 1 kHz was focused on the surface of a crystalline-silicon wafer, which was immersed in deionized water, by using a lens with a focal length of 150 mm. Once the ablation was completed, the aqueous solution containing silicon NSs was centrifuged with a speed of 12,000 rpm to separate silicon NSs with diameters of 150−250 nm from small silicon nanoparticles.

For the fabrication of silicon NPs on a quartz substrate, 220-nm-thick crystalline-silicon film was first transferred to a quartz substrate by adhesive wafer bonding. Then, a 180-nm-thick electron-beam photoresist (ZEP520A) was spin-coated on the surface of crystalline-silicon film and baked at 180 °C for 10 min. After that, a 50-nm-thick aluminum layer was deposited by thermal evaporation to avoid static charging during electron-beam lithography. The pattern was exposed by an electron beam writer (Vistec EBPG-5000plusES, Raith) at an acceleration voltage of 100 KeV. The aluminum layer was removed by using tetramethylammonium hydroxide and the photoresist was developed by using xylene. The pattern transfer was carried out by using inductively coupled plasma (Oxford Instruments) in HBr gas. Finally, the pattern resist was removed by using O_2_ plasma etching.

Silicon NPs can also be fabricated directly on a SOI wafer (SOITEC Inc.) with a 220-nm-thick silicon layer and a 2-μm-thick SiO_2_ layer. First, the pattern was defined on a positive electron beam resist film (ZEP 520A) with a thickness of 500 nm by using an electron-beam lithography tool (Raith 150 II) at an acceleration voltage of 30 KeV. Then, the pattern was transferred to the 220 nm SOI device layer by using an inductively coupled plasma etcher (PlasmaPro 100, Oxford) in the mixture of SF6 and C4F8 gas. The resist was then removed with acetone in ultrasonic cleaner followed by a washing process.

### Characterization of silicon nanoparticles

The morphologies of both silicon NSs and NPs were examined by scanning electron microscope (Ultra55, Zeiss) and transmission electron microscope (JEM-2100HR, JEOL) observations. Meanwhile, the nonlinear optical properties were characterized by using an inverted microscope (Axio Observer A1, Zeiss) equipped with a spectrometer (SR-500i-B1, Andor) and a charge-coupled device (DU970N, Andor). The femtosecond laser light (130 fs and 76 MHz) was focused on silicon NSs or NPs by using the 100× objective lens of the microscope and the generated nonlinear optical signals were collected by using the same objective lens and delivered to the spectrometer for analysis. The up-converted luminescence images for the arrays of silicon NPs were taken by a laser scanning confocal microscope system (LSM780 NLO, Zeiss) equipped with a tunable femtosecond laser (Chameleon ULTRA II, Coherent).

### Measurement of the luminescence decay time

We measured the luminescence decay time of silicon NSs based on the time-correlated single-photon counting technique by using a fluorescence lifetime spectrometer (LifeSpec-1400, Edinburgh Instruments) equipped with a microchannel plate photomultiplier (Hamamatsu). The luminescence decays were analyzed by using the F900 software package (version 7.1.3) and the decay times were extracted based on a reconvolution fitting analysis in which the instrumental response function was taken into account (see Fig. 3b, Supplementary Note 7, Supplementary Figs. 17, 23).

### Measurement of the quantum efficiency of single silicon NSs

We proposed a new method to characterize the quantum efficiency of the silicon NS (see Supplementary Note 3). The experimental setup used to characterize the nonlinear optical properties of the silicon NS was employed to measure the quantum efficiency of the silicon NS. Since the silicon NS was placed on a glass slide, the excitation laser light would be reflected by the glass slide, scattered by the silicon NS in both forward and backward directions, and absorbed linearly and nonlinearly by the silicon NS. A dichroic mirror with a narrow stop-band centered at ~786 nm and an optical density of 4 × 10^−6^ was used to reflect the excitation laser light. The excitation laser light reflected by the glass slide will be attenuated by a factor of 4 × 10^−6^ after passing through the dichroic mirror while the luminescence light outside the stop-band can fully transmit the dichroic mirror. In the experiment, we selected to resonantly excite a silicon NS with a MD resonance at ~786 nm.

From the nonlinear response spectra measured for the silicon NS at different excitation power densities, the attenuation coefficient of the measurement system was determined to be 5.67 × 10^−6^ at a low excitation power density of 5.10 × 10^−5^ W μm^−2^. In addition, the number of photons absorbed by the silicon NS at a high excitation power density of 2.95 × 10^−4^ W μm^−2^ was derived to be *N*_abs_ = 4.50 × 10^12^ (see Supplementary Note 3 for the details). Moreover, the number of photons emitted by the silicon NS was derived to be *N*_em_ = 1.84 × 10^10^ after considering the averaged collection efficiency of the objective over the entire emission spectrum (~12.8%). Therefore, the quantum efficiency for the photon to photon conversion of the silicon NS was estimated to be *η* ~0.409%. Considering that the nonlinear absorption was dominated by 3PA, the quantum efficiency for the conversion of the generated electron-hole pairs to the emitted photons was estimated to be *η* ~1.22%. Apart from the quantum efficiency of the silicon NS, the carrier density generated in the silicon NS was derived to be ~1.0 × 10^20^ cm^−3^ where a significant Auger recombination process is expected.

### Numerical modeling

The scattering spectra of silicon NSs and NPs were either analytically calculated based on Mie theory or numerically simulated by using the finite-difference time-domain technique. The multipolar contributions to the total scattering of the silicon NSs are calculated analytically^39^ while the corresponding results of the silicon NPs are evaluated numerically based on the decomposition utilizing spherical harmonics^40^. The permittivity of silicon was fitted from experimental data. In the finite-difference time-domain simulation, we employed a total-field/scattered-field source to evaluate the scattering efficiency of silicon NSs and NPs. A uniform mesh size with the smallest one of 1 nm was used to obtain converged simulation results and perfectly matched layer boundary condition was employed to terminate the finite simulation region. For the calculation of *I*, *I*^2^, and *I*^3^, the integrations of the electric field run over the entire volume of silicon NSs or NPs. We calculated the total collection efficiency of the emitted photons from a silicon NS by considering both the external quantum efficiency of the silicon NS (i.e., including the linear absorption of the silicon NS) and the collection efficiency of the 100× objective lens with a numerical aperture of 1.4. First, the directional emission efficiency of an ED emitter at a certain wavelength was calculated by averaging the directional emission intensities obtained at different locations inside the silicon NS. For each location, the averaged value for three orthogonal orientations of the ED emitter was derived. The wavelength-dependent total collection efficiency was obtained by using Fourier transform of the time domain signal under the excitation of a Gaussian pulse. The glass substrate supporting the silicon NS was also taken into account. Then, the collection efficiency of the objective lens was calculated by evaluating the near to far-field projection over a cone defined by the numerical aperture of the objective lens. We used the rotational symmetry of the system to reduce our computational load. The ED emitter is placed at a semicircle plane where the optical axis lies in. The wavelength-dependent total collection efficiency was shown in Supplementary Fig. 8. Finally, the wavelength-averaged total collection efficiency was estimated to be ~12.8%.

### Data availability

The data that support the findings of this study are available from the authors upon reasonable request.

## Electronic supplementary material

Supplementary Information
