# Supplementary material for: Lighting up silicon nanoparticles with Mie resonances
Source: Nat Commun. 2018 Jul 27;9:2964. doi: 10.1038/s41467-018-05394-z (PMC6063972; doi:10.1038/s41467-018-05394-z)
Supplement: Supplementary file 1 — Supplementary Information [file 41467_2018_5394_MOESM1_ESM.pdf]

Supplementary Information for 'Lighting up silicon nanoparticles with Mie resonances' by Zhang et al.

Supplementary Note 1. Transmission electron microscope image of the silicon nanosphere fabricated by femtosecond laser ablation

We fabricated silicon nanospheres (NSs) by using femtosecond laser ablation and regularly arranged silicon nanopillars (NPs) by using electron beam lithography and reactive ion etching. The crystalline phase of the silicon NSs was characterized by transmission electron microscopy. The transmission electron microscope image of a typical silicon NS is shown in Supplementary Figure 1. The crystalline phase of the silicon NS is indicated by paralleled red lines. There is a very thin amorphous shell on the surface of the silicon NS.

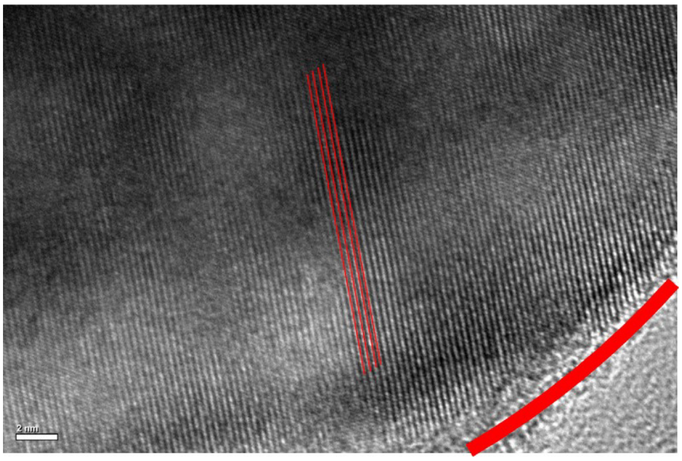

Supplementary Figure 1. Transmission electron microscope image of a typical silicon nanosphere fabricated by using femtosecond laser ablation. The crystalline phase of the silicon nanosphere is indicated by paralleled red lines while the outer surface is indicated by a bold red curve. The length of the scale bar is 2 nm.

Supplementary Note 2. Electric and magnetic resonances in silicon nanospheres

Based on Mie theory, the total scattering of a nanoparticle can be decomposed into the contributions of electric and magnetic resonances of different orders. As an example, we show the decomposition of the scattering spectrum of a silicon NS with d = 192 nm into the contributions of magnetic dipole (MD), electric dipole (ED), magnetic quadrupole (MQ), and electric quadrupole (EQ) resonances in Supplementary Figure 2. It is noticed that except the ED resonance all the resonances exhibit large quality factors. For example, quality factors of ~38 and ~27 are observed for the MQ and EQ resonances, implying that a large enhancement of the electric field can be achieved at these resonances. This feature indicates the possibility of realizing efficient excitation at the MD resonance and efficient up-converted luminescence at the MQ and EQ resonances.

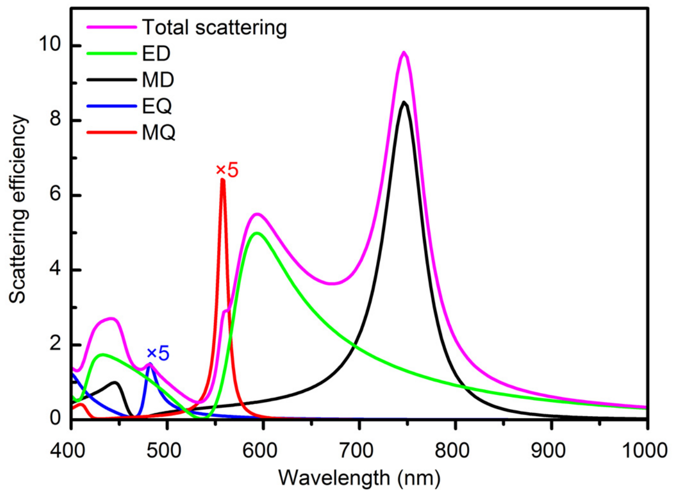

Supplementary Figure 2. Scattering spectrum of a silicon nanosphere calculated based on Mie theory. The diameter of the nanosphere is d = 192 nm. The contributions of magnetic dipole (MD), electric dipole (ED), magnetic quadrupole (MQ), and electric quadrupole (EQ) resonances to the total scattering are also presented.

Supplementary Note 3. Measurement of the quantum efficiency of a single silicon nanosphere

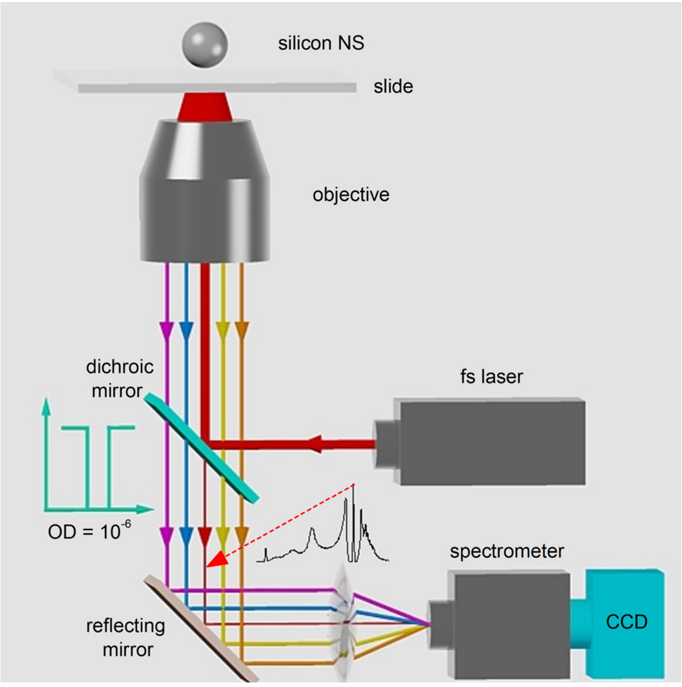

Supplementary Figure 3. Characterization of quantum efficiency. Schematic showing the experimental setup used to measure the quantum efficiency of a single silicon NS.

The measurement of the internal quantum efficiency of a single silicon nanoparticle is a big challenge from the technical point of view because of the unknown cross section of multiphoton-induced absorption. Therefore, the key point is to accurately determine the multiphoton-induced absorption of the silicon NS and the attenuation coefficient of the collected signal in the measurement system. We proposed a new method to characterize the quantum efficiency of a

single silicon NS by exploiting the nonlinear dependence of the multiphoton-induced absorption of the silicon NS on the excitation power density. The experimental setup used to measure the quantum efficiency of the silicon NS is schematically shown in Supplementary Figure 3.

As shown in Supplementary Figure 3, the excitation laser light was reflected by using a dichroic mirror and focused by using an objective lens on the silicon NS which was placed on a glass slide. The luminescence generated by the silicon NS and the excitation laser light reflected by the glass slide and scattered by the silicon NS in the backward direction were collected by the same objective lens and directed to a spectrometer for analysis after passing through the dichroic mirror. The reflectivity of the glass slide without the silicon NS was measured to be ~4.0% at the wavelength of the excitation laser light (~786 nm) and ~5.0% with the presence of the silicon NS, as shown in Supplementary Figure 4.

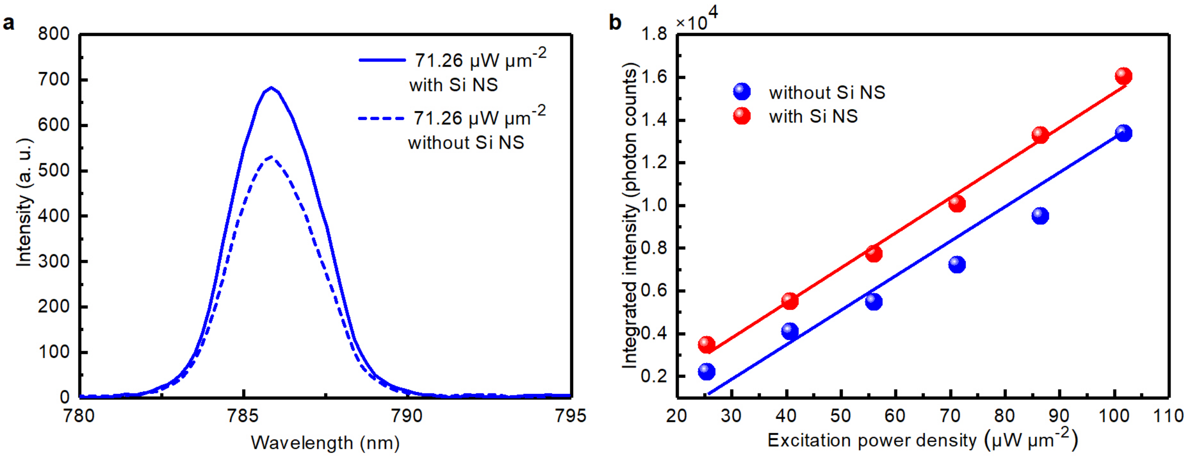

Supplementary Figure 4. Excitation power density dependent reflection measurements. (**a**) Spectra of the reflected laser light measured at low excitation power densities without (dashed curves) and with (solid curves) the presence of a silicon nanosphere (NS) with $d \sim 200$ nm. (**b**) Dependence of the integrated intensity of the reflected laser light on the excitation power density measured without and with the presence of the silicon NS.

In order to eliminate the reflected laser light, the transmission spectrum of the dichroic mirror was designed to possess a narrow stop-band centered at ~786 nm with an optical density (OD) of 4×10^{-6} and a bandwidth of ~40 nm. It means that the reflected laser light would be attenuated by a factor of 4×10^{-6} after passing through the dichroic mirror. Meanwhile, the luminescence outside the stop-band could completely go through the dichroic mirror and arrive at the detector. In the experiment, we selected a silicon NS whose MD resonance located at ~786 nm and excited it resonantly at the MD resonance. In this case, the energy of a single photon at the excitation wavelength is calculated to be ~1.58 eV. A 100× objective lens was used to focus the excitation laser beam to a spot of ~1.0 μm in diameter, corresponding to an area of ~0.785 μm^2.

Although a stop-band filter with an OD ~ 4×10^{-6} was used, we could still detect the reflected laser light in the nonlinear response spectra of the silicon NS, as shown in Supplementary Figure 5. Since the reflectivity of the glass slide and the linear scattering/absorption cross section of the silicon NS are independent of the excitation power density, it is expected that the intensity of the reflected laser light is linearly dependent on the excitation power density. This behavior was confirmed by examining the dependence of the reflected laser light intensity on the excitation power density, as shown in Supplementary Figure 6. However, this conclusion is valid only at low excitation power densities when the nonlinear absorption (i.e., the multiphoton-induced absorption) of the silicon NS can be neglected. At high excitation power densities when the nonlinear absorption becomes dominant, a deviation from the linear relationship (indicated by

the red line in Supplementary Figure 6), which is caused mainly by the three-photon-induced absorption (3PA), was observed. It means that the intensity of the reflected laser light, which has experienced the 3PA of the silicon NS, will become smaller than the value expected from the linear relationship, as shown in Supplementary Figure 6. Therefore, this unique feature makes it possible to evaluate the nonlinear absorption of the silicon NS at high excitation power densities where efficient white light emission is achieved.

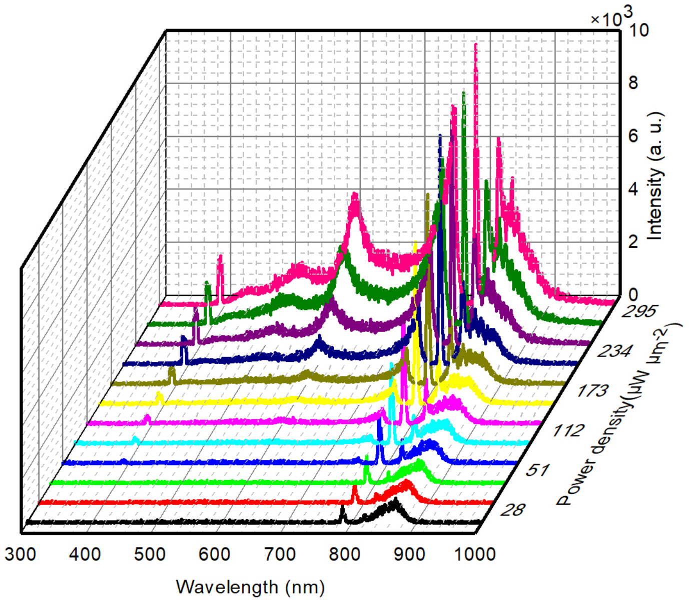

Supplementary Figure 5. Excitation power density dependent luminescence. Luminescence spectra measured for the silicon nanosphere (*d* ~200 nm) with magnetic dipole resonance at ~786 nm at different excitation power densities.

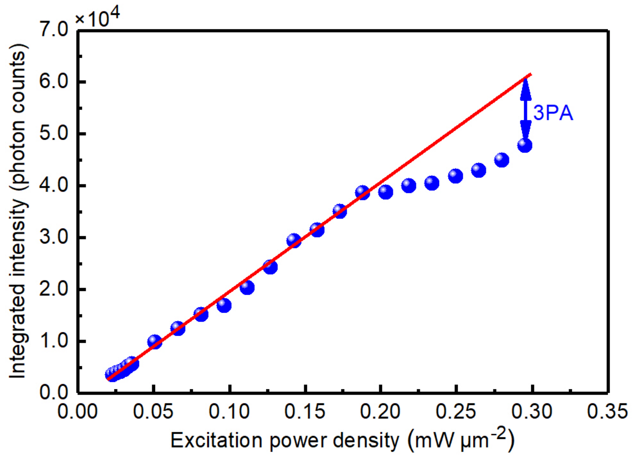

Supplementary Figure 6. Dependence of the integrated intensity of the reflected laser light on the excitation power density. It was derived for the silicon nanosphere (*d* ~ 200 nm) resonantly excited at the magnetic dipole resonance. The straight line shows the linear dependence observed at low excitation power densities when the nonlinear absorption is negligible.

We first estimated the attenuation coefficient of the measurement system (α) for the collected signals. At a low excitation power density of $P_1 = 5.10 \times 10^{-5}$ W μm^{-2} when the nonlinear absorption is negligible, the photon flux incident on the excitation spot (~0.785 μm^2) was calculated to be ~1.6×10^{14} s^{-1}. Such photons were firstly reflected by the glass slide with a reflectivity of r ~ 5.0% (with the presence of the silicon NS), and then attenuated by both the stop-band filter (OD = 4×10^{-6}) and the measurement system with a fixed attenuation coefficient (α), and finally amplified by the charge coupled device (CCD) with gain of g = 155 and quantum efficiency of ~ 0.70 at 786 nm. In this case, the total number of

photons recorded by the CCD within an exposure time of t = 0.5 s was found to be $N_1 \sim 9.87 \times 10^3$ (see Supplementary Figure 6). From the above result, the attenuation coefficient of the measurement system was determined to be $\alpha = 5.69 \times 10^{-6}$, which will be used in the following derivation of the quantum efficiency of the silicon NS.

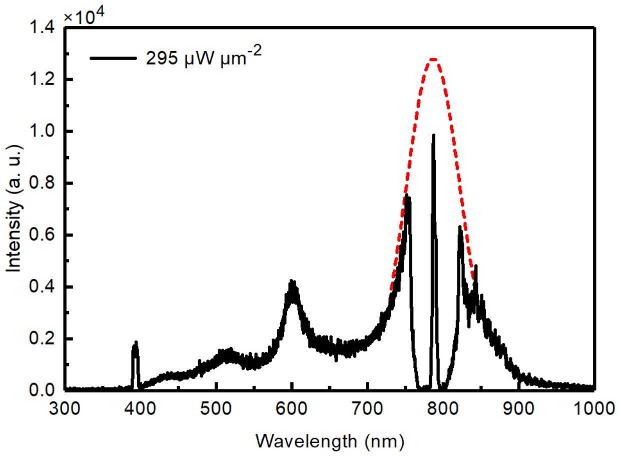

Supplementary Figure 7. Luminescence spectrum of a typical silicon nanosphere. Luminescence spectrum obtained at an excitation power density of 295 μW μm^{-2} for the silicon nanosphere ($d \sim 200$ nm) resonantly excited at the magnetic dipole resonance. The missing part of the spectrum within the stop band of the filter has been reconstructed by fitting the spectrum with multiple Lorentz line shapes (see red dashed curve).

After knowing the attenuation coefficient of the measurement system, we were able to estimate the quantum efficiency of the silicon NS at a high excitation power density of $P_2 = 2.95 \times 10^{-4}$ W μm^{-2} where efficient white light emission was observed, as shown in Supplementary Figure 7. From Supplementary Figure 6, the integrated intensity of the reflected laser light in terms of photon number expected at $P_2 = 2.95 \times 10^{-4}$ W μm^{-2} from the linear relationship was found to be $N_2 = 6.17 \times 10^4$. However, the number of photons detected by the CCD was only $N_2' = 4.78 \times 10^4$. The reduction in the photon number, which was caused by the 3PA of the silicon NS, was derived to be $\Delta N = 1.39 \times 10^4$. At the low excitation power density of $P_1 = 5.10 \times 10^{-5}$ W μm^{-2}, the number of photons reflected by the glass slide within the exposure time of the CCD (t = 0.50 s) was $N_0 = 4.0 \times 10^{12}$ because the incident photon flux was 1.6×10^{14} s^{-1} and the reflectivity of the glass slide (with the presence of the silicon NS) was ~5.0%. In this case, the number of photons detected by the CCD was $N_1 = 9.87 \times 10^3$. Therefore, the number of photons absorbed by the silicon NS at the high excitation power density of $P_2 = 2.95 \times 10^{-4}$ W μm^{-2} was derived to be $N_{abs} = (\Delta N/N_1) \times N_0 = 4.50 \times 10^{12}$.

Apart from the number of photons absorbed by the silicon NS, we have to find out the number of photons emitted by the silicon NS at $P_2 = 2.95 \times 10^{-4}$ W μm^{-2}. This can be done by integrating the luminescence spectrum (from 400 to 1000 nm) of the silicon NS shown in Supplementary Figure 7. Although the luminescence spectrum in the wavelength range of the stop band was removed, we could reconstruct the missing part by fitting it with multiple Lorentz line shapes, as shown in Supplementary Figure 7 (see the red dashed curve). In this way, the number of the emitted photons, which were finally detected by the CCD (g = 155, $\eta_{CCD} \sim 0.80$ in average), was estimated to be $N_{em}' = 1.65 \times 10^6$.

In order to derive the number of photons emitted by the silicon NS, we need to know the external quantum efficiency of the silicon (after taking into account the linear absorption of the silicon) and the collection efficiency of the 100× objective lens with numerical aperture of 1.4. Since the emission of the silicon NS originates from the electric dipole transition, the luminescence can be resembled by a large number of randomly distributed and oriented EDs in the silicon NS. At the same time, the radiation intensity and pattern of an ED, which influences the number of photons collected by the objective lens, depends strongly on the location and wavelength of the ED. For this reason, we have simulated

numerically the total collection efficiency of the emitted photons from the silicon NS, which includes the external quantum efficiency of the silicon and the collection efficiency of the 100× objective lens, by using the finite-difference-time-domain method (see Methods for the details) and obtained a wavelength-dependent total collection efficiency for the silicon NS, as shown in Supplementary Figure 8. As can be seen from this figure, a wavelength-averaged collection efficiency of $\beta \sim 12.8\%$ was obtained.

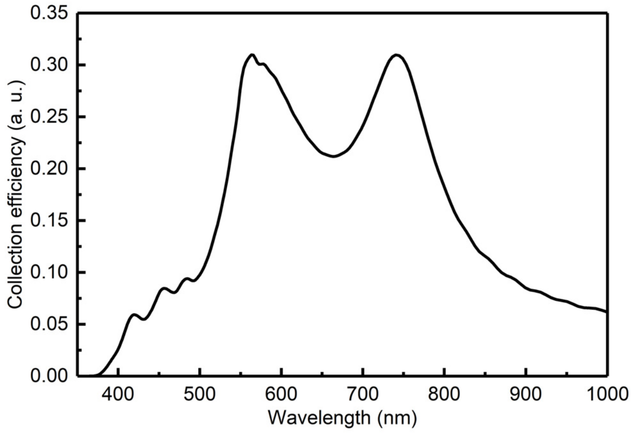

Supplementary Figure 8. Collection efficiency of the optical microscope. Wavelength-dependent total collection efficiency calculated for the silicon nanosphere (d ~192 nm) by using the finite-difference-time-domain simulation, after taking the external quantum efficiency of the silicon nanosphere and the collection efficiency of the 100× objective lens with numerical aperture of 1.4 into account.

Based on the total collection efficiency of the objective lens ($\beta \sim 12.8\%$), the attenuation coefficient of the measurement system ($\alpha = 5.69 \times 10^{-6}$), and the gain ($g = 155$) and quantum efficiency ($\eta_{CCD} \sim 0.80$ in the visible light region) of the CCD, the number of photons emitted by the silicon NS was eventually determined to be $N_{em} = N_{em}'/(\beta \times \alpha \times g \times \eta_{CCD}) = 1.84 \times 10^{10}$. Therefore, the quantum efficiency of the silicon NS was estimated to be $\eta = N_{em}/N_{abs} \sim 4.09 \times 10^{-3}$. This value gives the photon-to-photon conversion efficiency. Considering that the nonlinear absorption was dominated by 3PA, the conversion efficiency of electron-hole pairs to photon was calculated to be $\eta \sim 1.22$ %.

Apart from the quantum efficiency of the silicon NS, we can also derive the carrier density generated in the silicon NS based on the total number of photons absorbed by the silicon NS ($N_{abs} = 4.50 \times 10^{12}$). Keeping in mind that the total number of photons absorbed by the silicon NS was calculated for the exposure time of the CCD ($t = 0.5$ s) in which there are 38 M (i.e., 3.8×10^7) pulses, the number of photons absorbed for a single pulse was found to be 1.25×10^5. The volume of the silicon NS with a diameter of $d = 200$ nm was estimated to be 4.0×10^{-15} cm^{-3}. Considering that three photons create an electron-hole pair, the carrier density generated in the silicon NS by a single pulse was derived to be ~1.0×10^{20} cm^{-3}. Since Auger recombination rate is proportional to the cubic of carrier density, it is expected that the Auger recombination process will alleviate significantly the carrier relaxation process, enhancing the possibility to observe the effective radiative recombination at the EQ, MQ and MD resonances where the radiative recombination rates have been enhanced by the strong electric field inside the silicon NS, as schematically shown in Figs. 1b and c.

In the experiments, we have measured the quantum efficiencies of three silicon NSs with similar diameters (i.e., their MD resonances located at ~786 nm). The experimental results for the other two silicon NSs, including the luminescence spectra measured at different excitation power densities, the dependence of the integrated intensity of the reflected laser light on the excitation power density, and the reconstructed luminescence spectrum at the excitation power density where the quantum efficiency was estimated, are shown in Supplementary Figure 9 and 10. The quantum efficiency (i.e., the conversion efficiency of electron-hole pairs to photon) derived for these two silicon NSs were 0.274 %

and 0.485 %. Therefore, it can be seen that the quantum efficiency depends strongly on the quality of silicon NSs, the excitation wavelength, and the excitation power density.

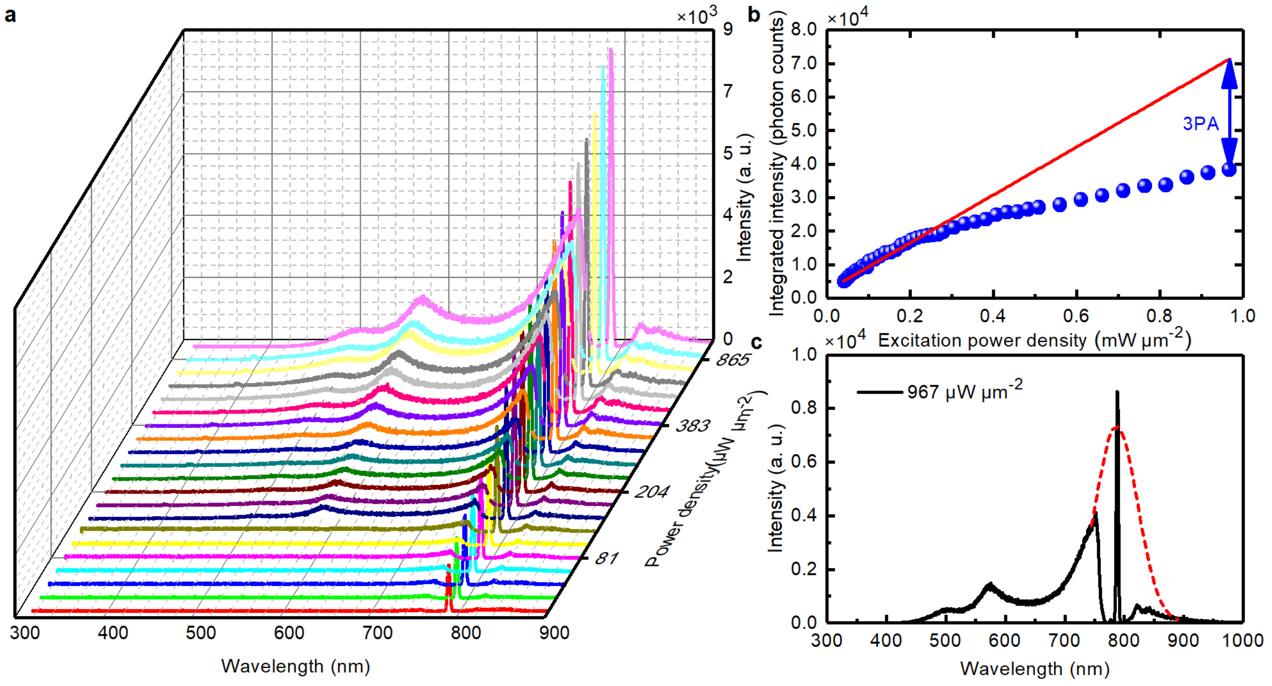

Supplementary Figure 9. Experimental results obtained for the second silicon nanosphere. (**a**) Luminescence spectra measured at different excitation power densities. (**b**) Dependence of the integrated intensity of the reflected laser light on the excitation power density. (**c**) Reconstructed luminescence spectrum measured at an excitation power density of 967 μW μm^{-2}.

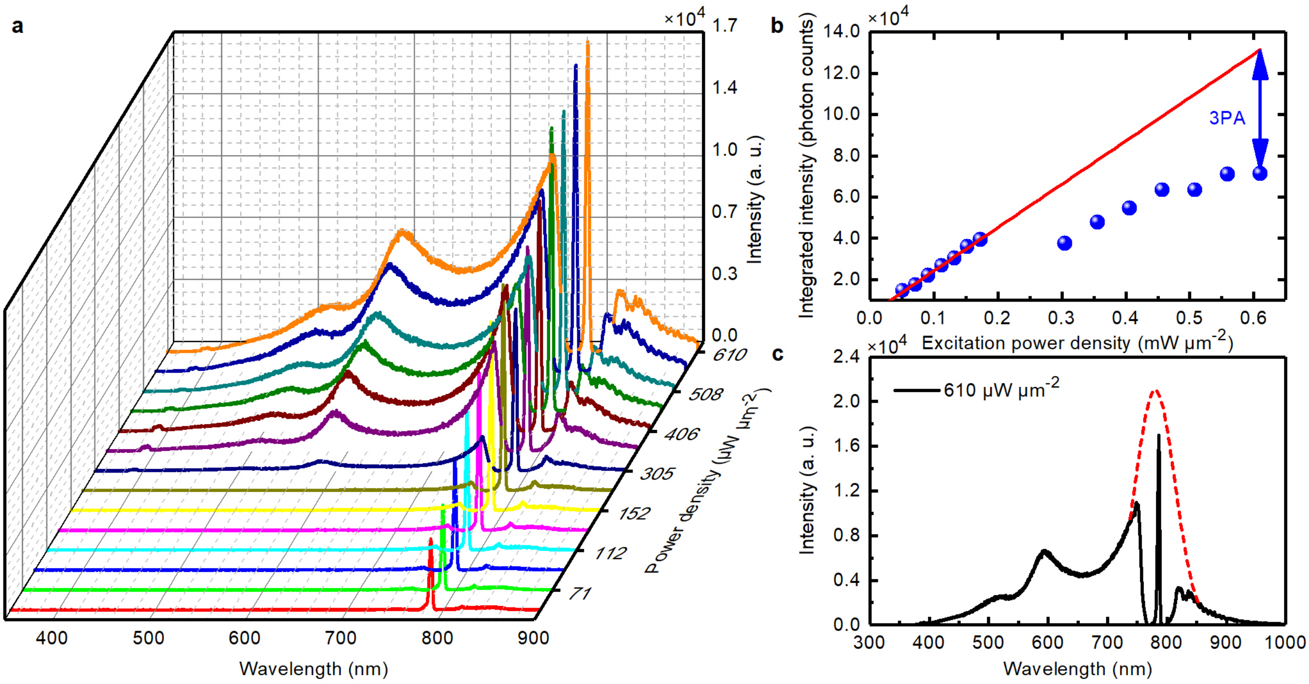

Supplementary Figure 10. Experimental results obtained for the third silicon nanosphere. (**a**) Luminescence spectra measured at different excitation power densities. (**b**) Dependence of the integrated intensity of the reflected laser light on the excitation power density. (**c**) Reconstructed luminescence spectrum measured at an excitation power density of 610 μW μm^{-2}.

In the measurements of quantum efficiency mentioned above, the MD resonances of the silicon NSs were located at ~786 nm and the luminescence was induced mainly by 3PA because the energy of two photons was smaller than the bandgap energy at the Γ point (~3.4 eV). For silicon NSs with MD resonances located at wavelengths shorter than 730 nm, the luminescence could be generated by 2PA through resonantly exciting the MD resonances. In this case, an enhancement of the luminescence intensity by more than one order of magnitude was observed, as shown in Supplementary Figure 11. An enhancement in the quantum efficiency is expected. Unfortunately, we do not have a dichroic mirror with a narrow stop band in this wavelength region to measure the quantum efficiency of the silicon NS.

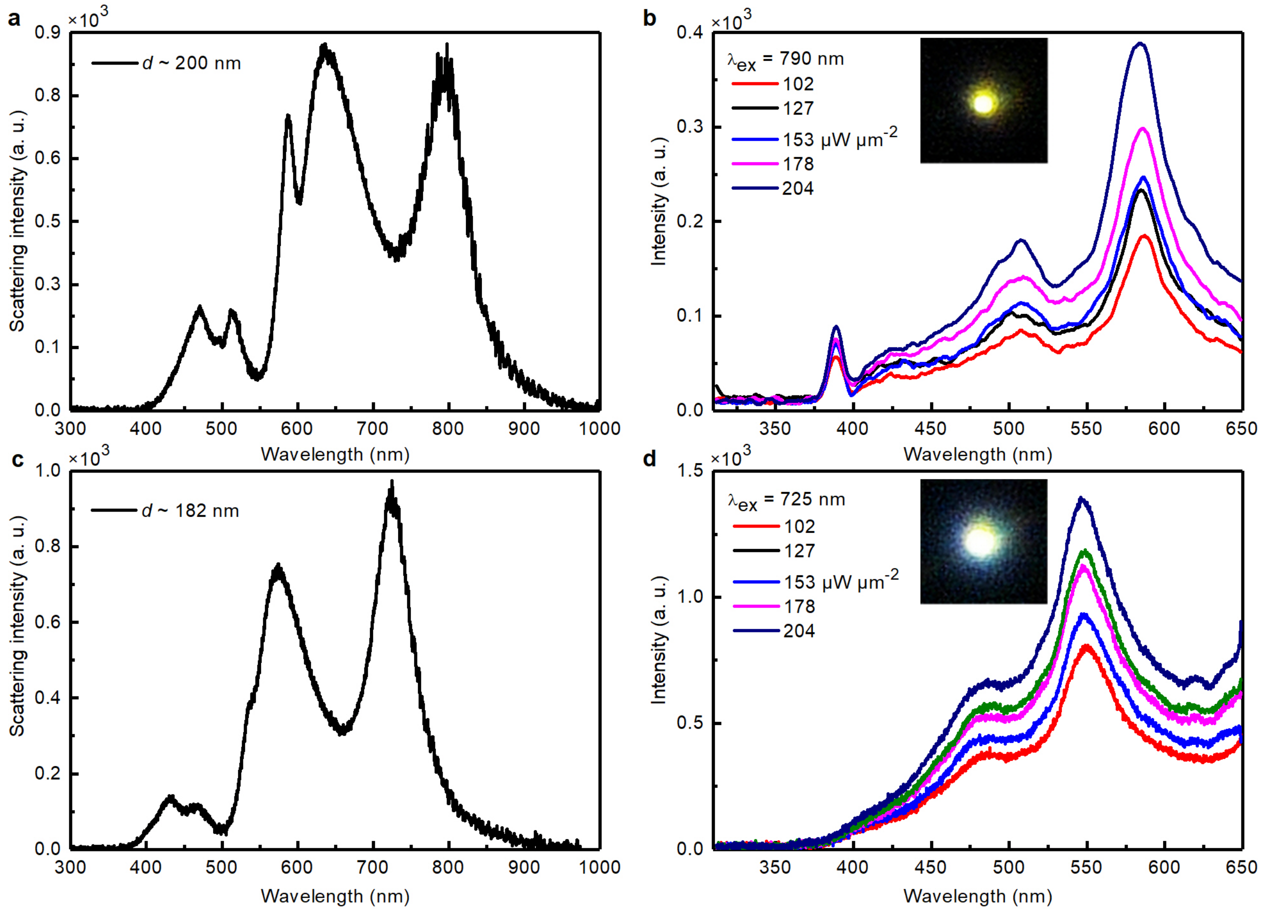

Supplementary Figure 11. Scattering and luminescence spectra measured for two silicon nanospheres with different diameters. Scattering spectra measured for silicon nanospheres with d ~ 200 nm (a) and d ~ 182 nm (c). The corresponding luminescence spectra measured by resonantly exciting the magnetic dipole resonances, which are located at ~790 nm and ~725 nm, are presented in (b) and (d), respectively. The charge coupled device images of the luminescence are shown in the insets.

Supplementary Note 4. Nonlinear response spectra and luminescence lifetimes of silicon nanospheres

We have examined the nonlinear optical responses of silicon NSs with different diameters of d = 103, 122 and 160 nm. The results measured at different pulse energies by using λ_{ex} = 800 nm are shown in Supplementary Figure 12. The images of the scattering light taken by using a CCD are presented in the insets, which appears to be blue, green and red respectively. In each case, it is noticed that the scattering spectrum is dominated by the MD resonance which is shifted to longer wavelengths with increasing the diameter of the silicon NS. For the silicon NS with d = 103 nm, only SHG was observed in the nonlinear response spectrum, as shown in Supplementary Figure 12b. In comparison, very weak up-converted luminescence was found at the long-wavelength side of the second harmonic for the silicon NS with d =

122 nm, as shown in Supplementary Figure 12d. For the silicon NS with d = 160 nm, it was observed that the intensity of the SHG decreased while that of the up-converted luminescence increased and became stronger than the SHG intensity, as shown in Supplementary Figure 12f. These results indicate clearly that the MD resonance of a silicon NS plays a crucial role in enhancing the up-converted luminescence.

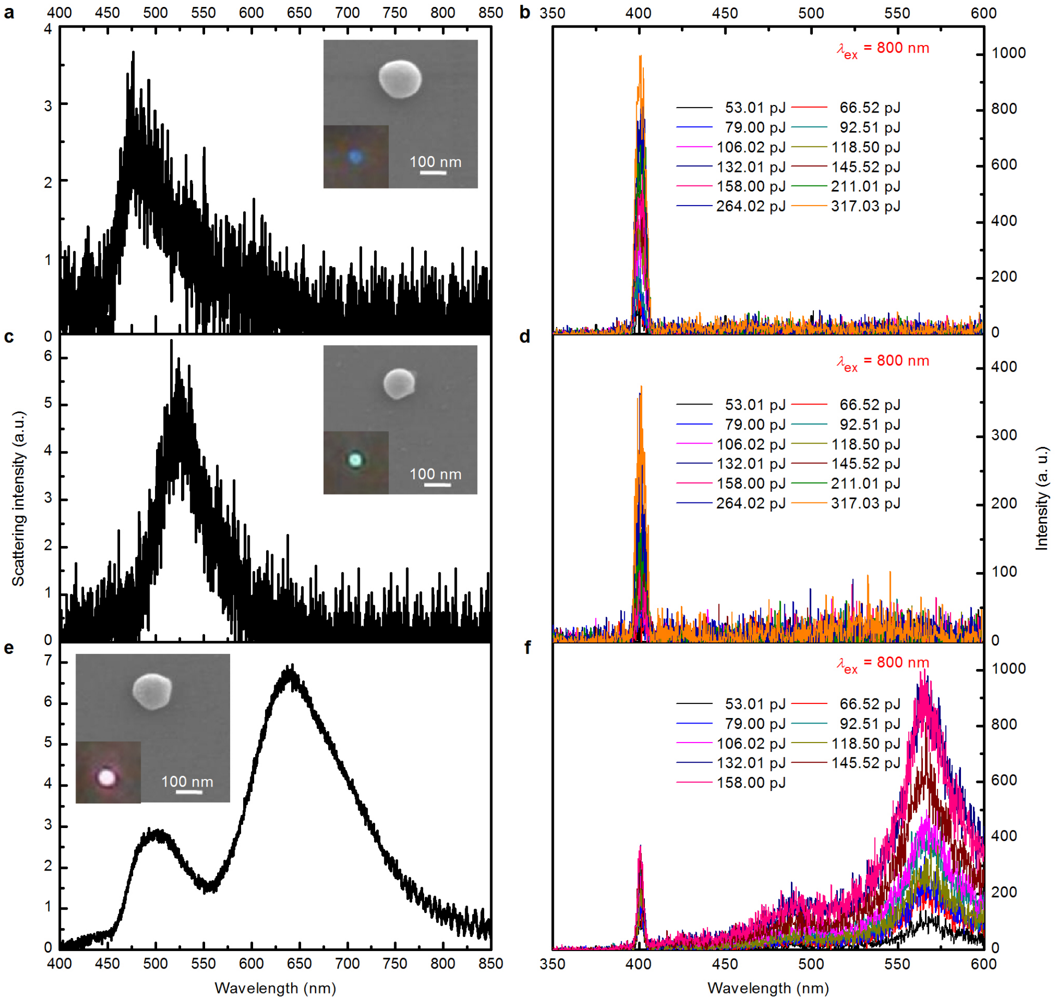

Supplementary Figure 12. Scattering and nonlinear response spectra measured for three silicon nanospheres with different diameters. Scattering spectra calculated and measured for a silicon nanosphere with d = 103 nm (**a**), d = 122 nm (**c**) and d = 160 nm (**e**). The scanning electron microscope images and charge coupled device images of the scattering light measured for the silicon nanospheres are shown in the insets. The nonlinear response spectra for the silicon nanospheres measured at different pulse energies are presented in (**b**), (**d**) and (**f**), respectively. The excitation wavelength was fixed at 800 nm.

In order to confirm that efficient white emission can be achieved by resonantly exciting the MD resonance of a silicon NS, we have measured the excitation spectra of two silicon NSs with similar diameters of $d \sim 200$ nm, as shown in Supplementary Figure 13. In both cases, it can be seen that the strongest luminescence intensity was observed when the wavelength of the excitation laser was chosen at the MD resonance of the silicon NS. A small deviation of the excitation wavelength from the MD resonance led to a rapid decrease of the luminescence intensity. This behavior is in good agreement with the wavelength dependence of the enhancement factor in electric field calculated for a silicon NS (see Fig. 1b).

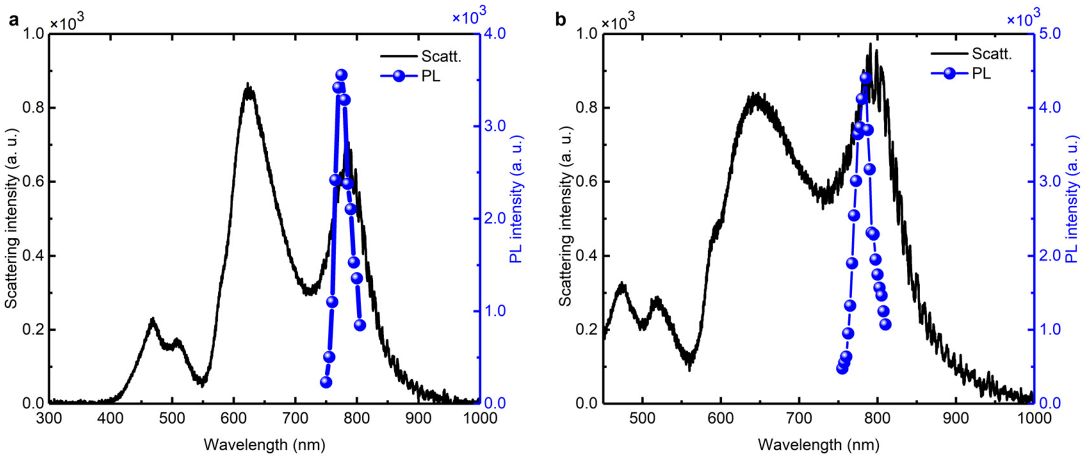

Supplementary Figure 13. Excitation spectra measurements. (**a**) and (**b**) Scattering and excitation spectra measured for two silicon nanoshperes with similar diameters of *d* ~ 200 nm, respectively.

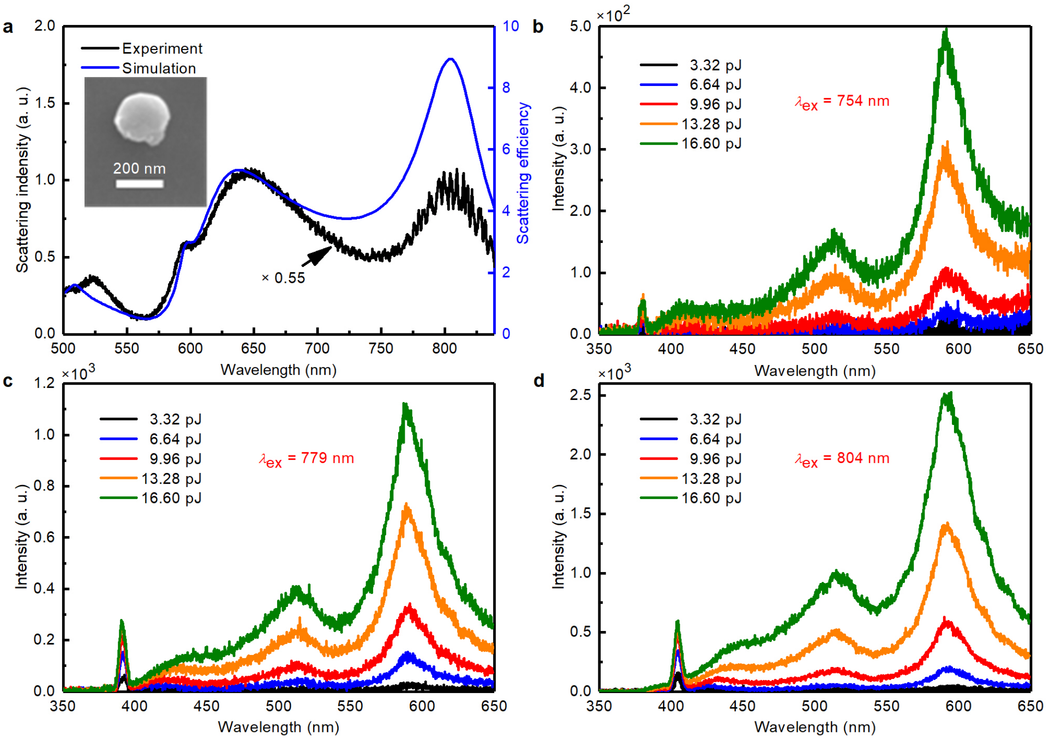

Supplementary Figure 14. Scattering and nonlinear response spectra measured for a typical silicon nanosphere. (**a**) Calculated and measured scattering spectra for a silicon nanosphere (NS) with d = 210 nm. The scanning electron microscope image for the silicon NS is shown in the inset. The nonlinear response spectra of the silicon nanoparticle measured at different excitation wavelengths of 754, 779, and 804 nm are shown in (**b**), (**c**), and (**d**), respectively.

In Supplementary Figure 14, we present the linear and nonlinear optical properties of another silicon NS with a larger diameter of d = 210 nm. Similar to the silicon NS whose linear and nonlinear properties are shown in Fig. 2, it is noticed that the up-converted luminescence of the silicon NS is also maximized when the MD resonance is resonantly excited.

The dependence of the up-converted luminescence on the excitation pulse energy of another silicon nanosphere is presented in Supplementary Figure 15. A slope equal to 2.9 was extracted from these data, indicating that the up-converted luminescence is dominated by 3PL.

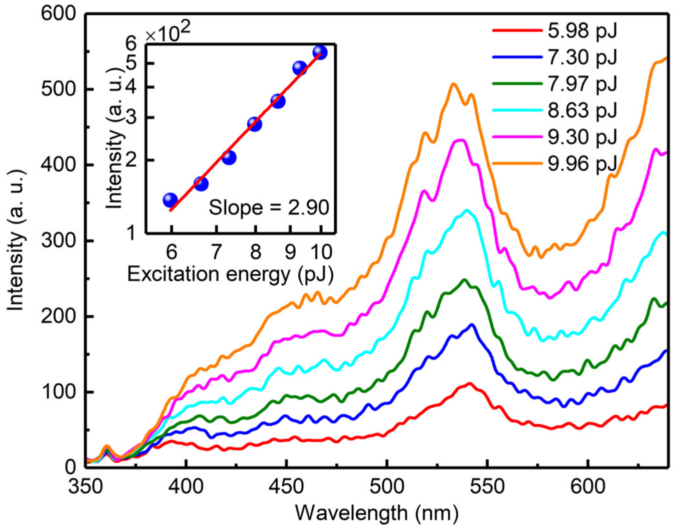

Supplementary Figure 15. Nonlinear response spectra of a silicon nanosphere. These spectra are measured for a silicon nanosphere with *d* ~ 190 nm at different pulse energies. The excitation wavelength was chosen to be 730 nm. The dependence of the luminescence intensity on the excitation pulse energy is shown in the inset.

In order to confirm that the hot luminescence from silicon NSs originates from the interband transition of hot carriers assisted by phonons, we calculated the dependence of the extracted slope on the wavelength (or energy) of the emitted photon, as shown in Supplementary Figure 16. For excitation wavelengths longer than 730 nm (see Supplementary Figure 16a-d), the extracted slope exhibited a constant close to 3.0 over the entire emission spectrum except at the EQ/MQ resonance where a reduced slope was observed. It indicates that the luminescence was induced mainly by 3PA. The reduced slopes observed at the EQ/MQ resonances imply enhanced photon conversion efficiencies in the up-converted luminescence at these wavelengths. For excitation wavelengths shorter than 730 nm (see Supplementary Figure 16e,f), a constant slope of ~1.5 was observed except the reduced slopes at the MQ and EQ resonances. In this case, the energy of two photons exceeded the bandgap energy at the Γ point and the luminescence was dominated by 2PA. This behavior is clearly distinct from the hot-electron intraband luminescence observed very recently in gallium arsenide NSs where a linear dependence of the extracted slope on the energy of the emitted photon was found [1]. The difference in the band structure between silicon and gallium arsenide is responsible for the distinct physical mechanisms of the hot luminescence observed in silicon and gallium arsenide NSs.

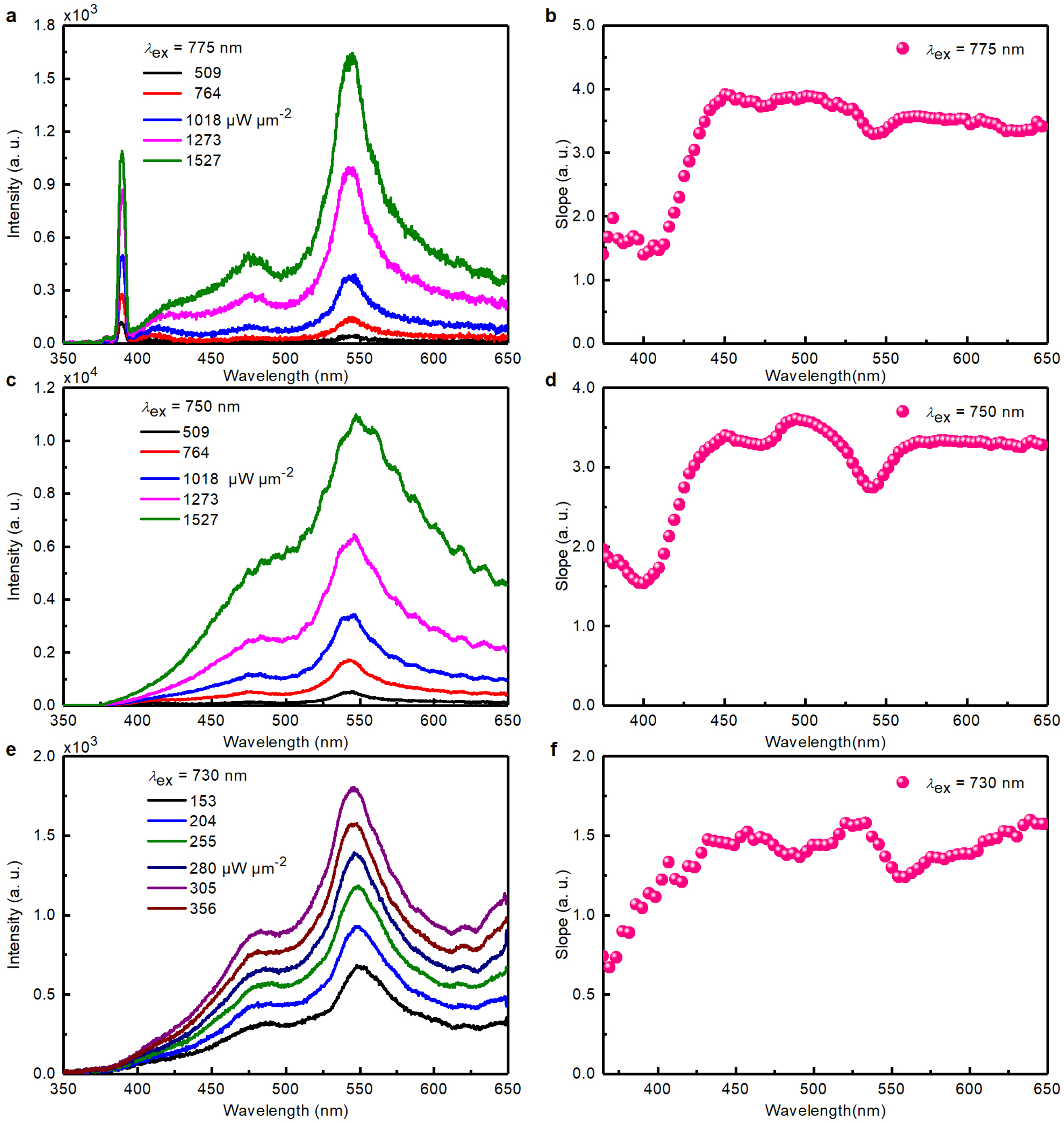

Supplementary Figure 16. Excitation power density dependent luminescence spectra and wavelength dependent extracted slopes measured for three silicon nanospheres. They were resonantly excited at their magnetic dipole resonances located at (**a**) 775 nm, (**c**) 750 nm, and (**e**) 730 nm. The corresponding dependences of the extracted slope on the wavelength of the emitted photon are shown in (**b**), (**d**), and (**f**), respectively.

In order to find out the relaxation time of the hot carriers which dominates the luminescence decay, we measured the luminescence lifetime for a Si NS with a diameter of $d \sim 190$ nm, as shown in Fig. 3b. The decay time extracted from a reconvolution fitting analysis was ~52 ps, after taking the instrument response function (IRF) into account.

We also measured the luminescence lifetimes for a few silicon NSs with different diameters. The decay times

extracted from the luminescence decays ranged from 32−52 ps, which is much longer than the relaxation time of hot carriers in bulk silicon (0.1−1.0 ps) due to the significantly enhanced Auger recombination rate (see Fig. 1c). The luminescence decay measured for the silicon NS with the smallest lifetime was shown in Supplementary Figure 17 from which the decay time was extracted to be ~32 ps.

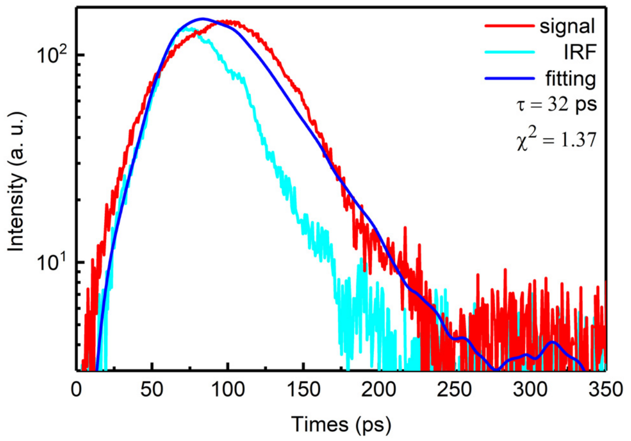

Supplementary Figure 17. Characterization of luminescence lifetime. Luminescence decay measured for a silicon nanosphere with a decay time of ~32 ps.

Supplementary Note 5. Nonlinear response spectra of silicon nanopillars fabricated on a silicon-on-insulator wafer

The scanning electron microscope images of the silicon NPs fabricated by using electron beam lithography in combination with reactive ion etching are shown in Supplementary Figures 18 and 19. In Supplementary Figure 20a, we show the scattering spectra calculated for two silicon NPs. One silicon NP has a diameter of d ~170 nm and the other is actually a truncated nanocone (NC) with diameters of d_1 = 210 and d_2 = 250 nm (see Supplementary Figures 18 and 19). It can be seen that the MD resonance of the silicon NP appears at ~790 nm while the ED resonance of the truncated silicon NC appears at ~760 nm (see Supplementary Figure 20a). In Supplementary Figure 20b, we also present the spectra of I^3 calculated for them and enhancement factors quite similar to those of the silicon NSs are observed, implying that efficient up-converted luminescence can also be achieved in silicon NPs if λ_{ex} is chosen at the MD or ED resonance. The nonlinear response spectra of the silicon NP resonantly excited at 790 nm are shown in Fig. 4a. Apart from the SHG appearing at 395 nm, it is noticed that the up-converted luminescence peaks are in good agreement with those predicted by numerical simulation (see Supplementary Figure 20). We also performed confocal microscopic imaging of the 3PL for the array of silicon NPs by using λ_{ex} = 790 nm and observed efficient up-converted luminescence from most silicon NPs in the array, as shown in the inset of Fig. 4a. One can find some silicon NPs in the array which didn't emit luminescence. It may be caused by the relatively large deviation of their sizes from the designed value, indicating the importance of resonant excitation in the generation of efficient up-converted luminescence. In Fig. 4b, we show the nonlinear response spectrum for the truncated silicon NC measured at different pulse energies and its corresponding confocal microscopic image. Differently, we chose to resonantly excite the ED resonance of the silicon NC by using λ_{ex} ~760 nm. Similar to the excitation of the MD resonance, the resonant excitation of the ED resonance of the silicon NC can also generate very efficient white light emission.

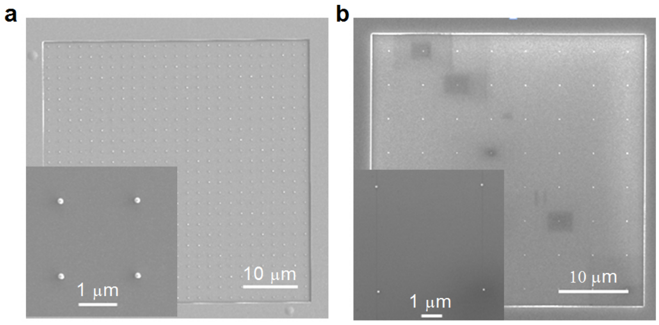

Supplementary Figure 18. Scanning electron microscope images of the silicon nanoparticles. (**a**) Scanning electron microscope image for the array of silicon nanoparticles with d = 170 nm, h = 220 nm, and p = 2 μm. (**b**) Scanning electron microscope image for the array of truncated silicon nanocone with d_1 = 210 nm, d_2 = 250 nm, h = 220 nm, and p = 5 μm. Here, p is the period of the array.

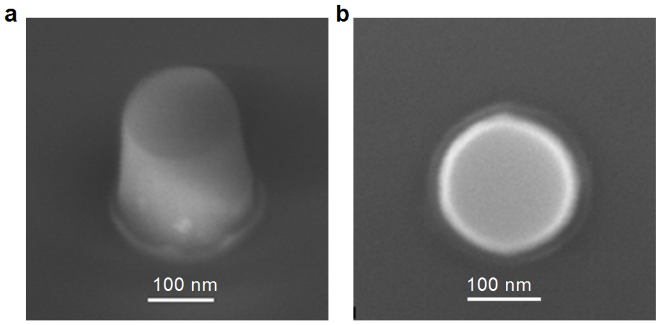

Supplementary Figure 19. Scanning electron microscope images for a typical truncated silicon nanocone used in the measurements of the nonlinear response spectra. (**a**) Side view. (**b**) Top view.

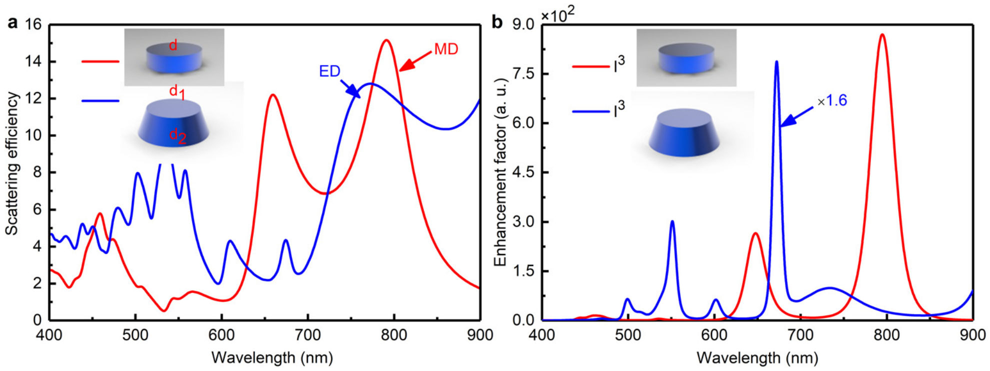

Supplementary Figure 20. Scattering efficiency and electric enhancement factor calculated for the structures used in Fig. 4 of the main text. (**a**) Scattering spectra calculated for a silicon nanoparticle with d = 170 nm and a truncated silicon nanocone with d_1 = 210 nm and d_2 = 250 nm. (**b**) Spectra of I^3 calculated for the silicon nanoparticle and truncated silicon nanocone.

Supplementary Note 6. Nonlinear response spectra of silicon nanopillars fabricated on a glass substrate

Since the substrate is not transparent for the silicon NPs fabricated directly on a silicon-on-insulator wafer, we cannot measure the scattering spectra of such silicon NPs by using our dark-field microscope. For this reason, the ED and MD resonances of the silicon NPs were determined only by numerical simulation according to the sizes of the silicon NPs estimated based on their scanning electron microscope images. In addition, it is difficult to accurately locate a single silicon NP without the help of bright field image in the measurements of up-converted luminescence, leading to a low excitation and collection efficiency. We then fabricated the silicon NPs on the transparent quartz substrate and studied

their nonlinear response under the excitation of femtosecond laser pulses, as shown in the following.

By utilizing spherical harmonics, the total scattering of a silicon NP can be decomposed into the contributions of electric and magnetic resonances of different orders. In Supplementary Figure 21, we show the decomposition results (up to quadrupole) for the scattering spectrum of a silicon NP with d = 148 nm and h = 220 nm. It is noticed that MD and ED resonances dominate the scattering process.

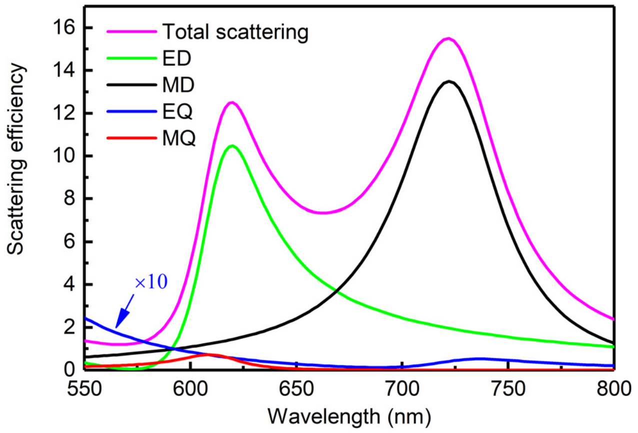

Supplementary Figure 21. Scattering spectrum of a silicon nanoparticle with d = 148 nm and h = 220 nm calculated by using finite-difference-time-domain simulation. The contributions of electric dipole (ED), magnetic dipole (MD), electric quadrupole (EQ), and magnetic quadrupole (MQ) resonances to the total scattering are evaluated numerically utilizing spherical harmonics.

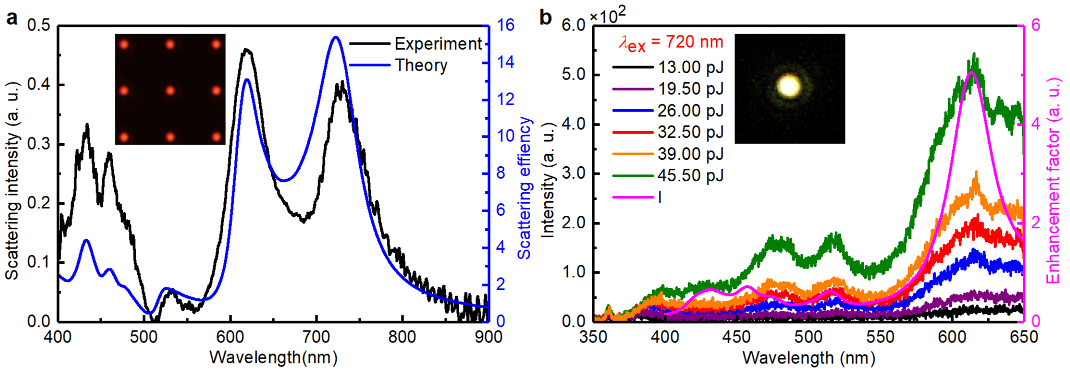

Supplementary Figure 22. Scattering and nonlinear response spectra of silicon nanoparticles. (**a**) Scattering spectra calculated and measured for a single silicon nanoparticle in a regular array of silicon nanoparticles on a quartz substrate whose dark-field microscope image is shown in the inset. The diameter, height and period of the silicon nanoparticle were estimated to be ~148 nm, ~220 nm and ~5 μm, respectively. (**b**) Nonlinear response spectra measured for the silicon nanoparticle resonantly excited at its magnetic dipole resonance (~720 nm) with different pulse energies. Spectrum of I calculated for the silicon nanoparticle is also provided. The inset shows the charge coupled device image of the up-converted luminescence.

In Supplementary Figure 22a, we show the scattering spectra measured and calculated for a single silicon NP in a regular array of silicon NPs fabricated on a quartz substrate (see Methods for details). The diameter, height and period of the silicon NP were estimated to be ~148 nm, ~220 nm and ~5 μm, respectively. It can be seen that the MD (~720 nm) and ED (~620 nm) resonances dominate the scattering spectrum of the silicon NP. The nonlinear response spectra of the silicon NP resonantly excited at 720 nm are shown in Supplementary Figure 22b. Similar to case of silicon NSs, the resonant excitation of the MD resonance of the silicon NP can also generate very efficient white light emission. Apart

from the SHG appearing at 360 nm, it is noticed that the peaks of the up-converted luminescence are in good agreement with those predicted by the numerical simulation (Supplementary Figure 22b).

Supplementary Note 7. Measurement of the luminescence decay times of silicon nanospheres and gallium arsenide wafer

In experiments, we measured the luminescence decay time by using the time-correlated single photon counting technique by using a fluorescence lifetime spectrometer (LifeSpec-1400, Edinburgh Instruments) equipped with a microchannel plate photomultiplier (MCP-PMT, Hamamatsu). The luminescence decays were analyzed by using the F900 software package (version 7.1.3) and the decay times were extracted based on a reconvolution fitting analysis after taking the instrument response function (IRF) into account.

The measured decay times ranged from 32−52 ps which is much shorter than the radiative lifetime of the hot carriers (~ 1−10 ns), implying that the luminescence decay in silicon NSs was dominated by the relaxation time of hot electrons from the Γ valley to the Δ valley in the band structure of silicon (see Fig. 1c). For safety, we also measured the luminescence decay of a gallium arsenide wafer (bulk gallium arsenide), the observed decay time of ~342 ps is in good agreement with that reported in literature, as shown Supplementary Figure 23.

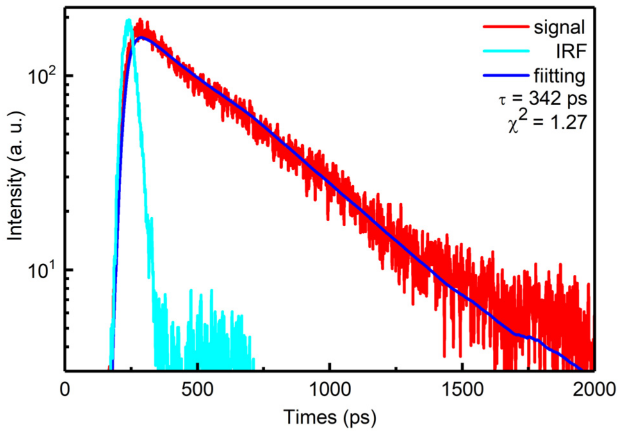

Supplementary Figure 23. Luminescence decay measured for bulk gallium arsenide. The decay time was extracted to be ~342 ps.

Supplementary References:

1. Haug, T., Klemm, P., Bange, S. & Lupton, J. M. Hot-electron intraband luminescence from single hot spots in noble-metal nanoparticle films. *Phys. Rev. Lett.* **115**, 067403 (2015).
